# The Role of Affect in Attentional Functioning for Younger and Older Adults

**DOI:** 10.3389/fpsyg.2012.00311

**Published:** 2012-08-31

**Authors:** Soo Rim Noh, Mary Jo Larcom, Xiaodong Liu, Derek M. Isaacowitz

**Affiliations:** ^1^Department of Psychology, Duksung Women’s UniversitySeoul, South Korea; ^2^Department of Psychology, Brandeis UniversityWaltham, MA, USA; ^3^Department of Psychology, Northeastern UniversityBoston, MA, USA

**Keywords:** affect, age differences, attentional networks, individual differences, attention

## Abstract

Although previous research has shown that positive affect (PA) and negative affect (NA) modulate attentional functioning in distinct ways, few studies have considered whether the links between affect and attentional functioning may vary as a function of age. Using the Attention Network Test (Fan et al., 2002), we tested whether participants’ current state of PA and NA influenced distinct attentional functions (i.e., alerting, orienting, and executive attention) and how the relationships between affective states and attentional functioning differ in younger (18–25 years) and older (60–85 years) age groups. While there were age differences in alerting efficiency, these age differences were mediated by PA, indicating that the higher state PA found in older adults may contribute to age differences in alerting. Furthermore, age group moderated the relationship between PA and orienting as well as NA and orienting. That is, higher levels of PA and lower levels of NA were associated with enhanced orienting efficiency in older adults. Neither PA nor NA had any influence on executive attention. The current results suggest that PA and NA may influence attentional functioning in distinct ways, but that these patterns may depend on age groups.

## Introduction

According to Larsen (2000), *affect* is the evaluative “feeling tone associated with mood and emotion” that is “felt as good or bad, as pleasant or unpleasant, as a felt tendency to approach or avoid” (p. 130). Affective states can be categorized into positive affect (PA) and negative affect (NA), and can influence on attentional processing (e.g., Fredrickson, 2004; Bless and Fiedler, 2006; Forgas, 2008). Advancing age is associated with emotional well-being, characterized by more positive emotional experience (decreased NA and increased or continuing PA) than found among younger adults (e.g., Mroczek and Kolarz, 1998; Charles et al., 2001). This age-related emotional experience led researchers to investigate the link between emotional aging and selective attention to affective content (e.g., Carstensen and Mikels, 2005). Few studies, however, have examined how age-related differences in affective experience influence multiple aspects of attention, such as alerting, orienting, and executive attention (e.g., Posner and Petersen, 1990). This is a critical goal of the current study because emotional factors may differentially influence theses aspects of attention (e.g., Moriya and Tanno, 2009; Jiang et al., 2011), and as suggested by age-related differences in affective experience, this may vary by age (Phillips et al., 2002b).

Attention is not a unitary function, but it encompasses multiple functions (e.g., Posner and Petersen, 1990; Fan et al., 2005; Posner and Rothbart, 2007). Posner and Petersen (1990) have distinguished three anatomically distinct attentional networks that serve different attentional functions: alerting, orienting, and executive attention. *Alerting* is defined as the ability to achieve and maintain an alert state, and facilitates response readiness for an incoming stimulus. *Orienting* refers to the ability to select and shift attention toward the location of an incoming stimulus. *Executive attention* involves the ability to resolve conflicts among various competing responses, thus the capacity to select relevant information and ignore irrelevant information. The Attention Network Test (ANT), a combination of Posner’s (1980) spatial cuing task and Eriksen and Eriksen’s (1974) flanker task, was developed to simultaneously assess the efficiency of each of the three attentional networks (Fan et al., 2002). In the ANT, the alerting effect is assessed by comparing RTs for targets preceded by alerting (warning) cues informing the temporal onset of the target with those not preceded by any cue. The orienting effect is assessed by comparing RTs for spatially cued targets with RTs for neutrally cued targets, and the orienting response is elicited via a peripherally presented cue without moving the eyes; therefore, *covert* orienting is measured in the ANT (Fernandez-Duque and Posner, 2001). Executive attention is assessed by comparing RTs for targets flanked by congruent distractors with those flanked by incongruent distractors (i.e., the conflicting effect). Behavioral studies using the ANT showed that estimates of efficiency in alerting, orienting, and conflict resolution were uncorrelated (e.g., Fan et al., 2002; Waszak et al., 2010). Moreover, neuroimaging studies further demonstrated mappings between these effects and three separate anatomical systems associated with each network (despite shared common activation sites among the networks; Fan et al., 2003, 2005; Posner and Rothbart, 2007, for a review). For example, the alerting network has been related to activation of the right frontal cortex and parietal region, while orienting network has been associated with posterior brain areas, including the superior parietal lobe, temporal parietal junction, and the frontal ocular fields. Executive attention is associated with the anterior areas of the frontal cortex (see Posner and Rothbart, 2007, for a review).

Operating a motor vehicle is one example of a daily task for many adults which requires effective functioning of the three attentional networks. For example, someone with good alerting may be better able to maintain a vigilant state while driving, being aware of *when* important information such as road signs, obstructions, and other vehicles appear. People who have high orienting functioning may be more sensitive to detect the direction from *where* that outside information is coming. The ability to effectively orient attention could potentially make the difference between avoiding and hitting objects that come into the path of the moving vehicle. Good executive attention is essential for negotiating complex traffic situations. Making the correct judgments and determining what environmental information is relevant in helping to make those judgments and what is not, is critical for safe driving. Because of age-related declines in sensory and motor systems (Seidler et al., 2010), good attentional functioning while driving may be even more critical for older individuals. Indeed one study found poorer performance on an executive functioning measure among those older adults who had been in an auto accident as compared to those who had not (Daigneault et al., 2002).

Aging has been associated with differential effects on the three attentional networks. There is evidence that healthy aging is associated with decreased alerting efficiency (Jennings et al., 2007; Isaacowitz et al., 2008; Gamboz et al., 2010). Age-related changes in the noradrenergic system, which make it difficult for older adults to maintain a vigilant state, have been attributed to age-related declines in alerting functioning (Jennings et al., 2007). On the other hand, no reliable age differences in orienting or executive attention networks were reported (e.g., Festa-Martino et al., 2004; Jennings et al., 2007; Gamboz et al., 2010), although a decline in neural functioning of these networks was reported (Lorenzo-Lopez et al., 2002; West and Moore, 2005). One study by Mahoney et al. (2010), which included a wide range of older adults, found that increasing age is associated with decreased executive attention efficiency. The findings on age differences in executive attention may support the recent argument by Verhaeghen (2011) that age-related deficits in executive control are overstated, as executive control related to resistance to interference (often measured by Flanker and Stroop tasks) shows a lack of age-related declines.

It has been well-demonstrated that individuals with mood disorders such as anxiety and depression show attentional bias toward negative information as these individual have difficulty disengaging attention from such stimuli (e.g., Koster et al., 2005; Mogg et al., 2008). Similarly, PA has been associated with attentional bias toward reward-related stimuli (Tamir and Robinson, 2007). In addition to attentional biases to emotional information modulated by PA or NA, affect also influences multiple aspects of attention that involve non-emotional information.

According to the Broaden-and-Build Theory (Fredrickson, 1998, 2001, 2004; Isen, 1999), PA expands or broadens attentional scope, leading to more creative and flexible thinking (Ashby et al., 1999). The down side of this broadening effect is that PA has been linked to impaired executive attention, as PA increases inhibition costs (Biss et al., 2009). PA has been associated with a larger flanker interference (Rowe et al., 2007) or a larger Stroop interference (Phillips et al., 2002a), impaired planning (Phillips et al., 2002b) and greater priming for distraction (Biss et al., 2009; Biss and Hasher, 2011). There is also evidence that PA enhances the rapid covert orienting of attention (Compton et al., 2004), but this PA modulation was found in individuals with low PA. Compton et al. (2004) interpreted their findings as showing “a corresponding lack of flexibility in attentional processing associated with low PA” (p. 741), which may increase more attentional bias toward a cued location. In contrast to the link between PA and attention, NA has been linked to the narrowing scope of attention (Fredrickson and Branigan, 2005), focusing more on details of a stimulus or the environment (Forgas, 1999). For example, Vermeulen (2010) has shown that NA increased inhibitory responses to task-irrelevant stimuli, while PA decreased the inhibition of distractors.

Relatively few studies have examined the link between affective states and different functions of attention, using the ANT (e.g., Moriya and Tanno, 2009; Pacheco-Unguetti et al., 2010; Jiang et al., 2011; Lyche et al., 2011). Across these studies, NA was associated with enhanced alerting (Pacheco-Unguetti et al., 2010; Jiang et al., 2011; Lyche et al., 2011; cf., Moriya and Tanno, 2009). This pattern is consistent with the idea suggesting that NA modulates the activation of noradrengeric systems, which maintain vigilant attention (Sullivan et al., 1999; Jiang et al., 2011). The studies yielded mixed results regarding the relationship between NA and orienting. Pacheco-Unguetti et al. (2010) showed that NA (particularly an anxious state) was related to enhanced alerting and orienting, while Moriya and Tanno (2009) found NA (state and trait anxiety, and depression) associated with decreased orienting efficiency (Moriya and Tanno, 2009). On the other hand, some studies found no association between NA and orienting (Finucane et al., 2010; Jiang et al., 2011; Lyche et al., 2011). These mixed findings may be attributable to method variance in the diverse ways affect is assessed; in these studies, individual differences in affect is assessed by self-reported questionnaires, or by experimentally induced mood procedures.

Investigations into the link between PA and attentional networks are limited (Finucane et al., 2010; Jiang et al., 2011). The few existing studies reported no association between PA and any of the three networks, although one might expect that PA may be associated with orienting (Compton et al., 2004) and executive attention (e.g., Phillips et al., 2002a; Rowe et al., 2007; Biss and Hasher, 2011). Nevertheless, the association between PA and attentional networks has not been significantly explored yet; therefore, it seems to be important to further examine the effect of PA on the functions of attentional networks.

Findings that state affect influences attention raises questions about the attentional functions of individuals who tend to experience certain affective states more frequently than others. A large body of literature indicates that older adults report experiencing more PA and less NA than their younger counterparts (e.g., Mroczek and Kolarz, 1998; Charles et al., 2001; Carstensen et al., 2011). According to socioemotional selectivity theory (SST; Carstensen and Turk-Charles, 1994; Carstensen et al., 1999), such positive emotional experiences are attributable to limited future time perspective that leads older adults (and others facing similar time horizons) to prioritize goals related to emotion regulation over other goals. Such motivational shifts, occurring with age, have been attributed what has been labeled the *positivity effect* when processing emotional information displayed by older adults. The positivity effect is a pattern of preferential processing of positive information over negative information (e.g., Carstensen and Mikels, 2005). A number of studies using eye-tracking to measure gaze patterns occurring during the processing of emotional stimuli, have demonstrated that older adults, compared to their younger counterparts, showed looking preferences toward positive and away from negative stimuli (when the positive or negative stimulus was paired with neutral stimuli; Isaacowitz et al., 2006; Isaacowitz and Noh, 2011, for a review). Indeed, older adults’ positive gaze patterns have been directly linked to their attempt to regulate emotion. Older adults commonly display mood-incongruent gaze patterns such that, in a negative mood state, they displayed more positive gaze preferences than those in a positive or neutral mood state. In the same task, younger adults showed more mood-congruent gaze patterns, such that their gaze preferences reflected their mood states (i.e., a more positive gaze pattern when in good moods, a more negative gaze pattern when in bad moods; Isaacowitz et al., 2008). Parallel to these behavioral findings, at the neural level, amygdalar engagement was equivalent among both younger and older adults (the amygdalar is the key structure responsible for processing of emotional information; Forgas, 2008); however, older adults have shown enhanced recruitment of dorsolaterral prefrontal regions (DLPFC) during encoding of negative stimuli (St Jacques et al., 2010). DLPFC is known as the key structure responsible for cognitive control of emotion (for reviews see Vuilleumier and Huang, 2009; Dolcos et al., 2011). Therefore, greater frequency of activation of the DLPFC may explain the increased motivation found among older adults to regulate emotion generated by negative stimuli (St Jacques et al., 2010; Dolcos et al., 2011).

Despite the growing knowledge linking the DLPFC, amygdalar activation, and emotion regulation, it remains unclear whether there are age differences in multiple aspects of attention as function of PA and NA when processing non-emotional information. A study by Phillips et al. (2002b) examined whether there were age differences in the effects of induced positive and negative mood on executive attention (i.e., planning). They found that older adults in both positive and negative mood states showed greater impairment in a planning task than younger adults. The authors interpreted this results as evidence that experiencing emotionally salient events before tasks may have more adverse effects on executive attention in older adults. There is also indirect evidence that older adults with good alerting and executive attention efficiency were more likely to rely on attentional strategies (i.e., attending to positive information) in order to regulate their emotions (Isaacowitz et al., 2009; Noh et al., 2011). It therefore seems reasonable to expect that there may be age differences in how affect influences the fundamental processes by which attention operates.

The current study aimed at investigating age differences in the relationship of state PA and NA to attentional networks using the ANT. While state PA and NA and trait PA and NA are related (Watson and Clark, 1984), focusing on state PA and NA allowed us to determine how potentially modifiable state affect can impact the functioning of the attentional networks. Moreover, given age-related changes in affective states (e.g., Mroczek and Kolarz, 1998), examining the link between state affect, and attentional networks across age groups may increase variability of state PA and NA, and thus could help resolve past mixed findings on this link. We hypothesized that PA and NA may exert differential effects on attentional networks and the pattern would vary by age groups (Phillips et al., 2002b). For alerting, we predicted that NA would be associated with enhanced alerting efficiency (e.g., Pacheco-Unguetti et al., 2010; Jiang et al., 2011). For orienting, PA would be associated with diminished orienting efficiency (Compton et al., 2004). NA may also be associated with orienting efficiency; however, previous findings on this subject are somewhat conflicting. In light of these mixed results, the current researchers feel it is important to further examine the link between NA and orienting in the current study. For executive attention, PA would be associated with decreased executive attention efficiency (Phillips et al., 2002a; Rowe et al., 2007). Although studies using the ANT found a lack of evidence for linking PA and executive attention, investigations were limited; therefore, it seems to be reasonable to expect such an association given previously reported findings (e.g., Phillips et al., 2002a; Rowe et al., 2007). With regard to age differences, we made three predictions. First, despite age-related declines in alerting efficiency (Jennings et al., 2007), we anticipated that older adults in a higher NA state would be more alert than those with in a lower NA state, as older adults with higher NA are motivated to regulate their emotion, which increases vigilance. Second, in light of findings that link older adults’ selective attention toward positive information to their mood states (Isaacowitz et al., 2008, 2009; Noh et al., 2011), we hypothesized that older adults’ orienting efficiency would be more likely to be influenced by their affective states than younger adults. In particular, NA would generate more efficient orienting, even for non-emotional processing. Although the ANT measures covert orienting while previous eye-tracking studies (showing age-related positive gaze preferences as a function of mood states) measured *overt* orienting attention (Isaacowitz et al., 2008, 2009; Noh et al., 2011), it has been suggested that both types of attentional orienting share the same neruroanotomical substrates (Fernandez-Duque and Posner, 2001). Third, older adults in both PA and NA states would show diminished executive attention as demonstrated by Phillips et al. (2002b).

## Materials and Methods

### Participants

Seventy-six younger adults (44 female; aged 18–25 years) and 69 older adults (53 female; aged 60–85 years) participated in the current study. Younger participants were recruited from an introductory psychology course and flyers posted on campus. Older adults were recruited from a lifelong learning program. Participants received either course credit or monetary stipend. The data for the current study come from a larger study that looked age differences in gaze preferences (Isaacowitz et al., 2008). An additional one younger and two older adults were tested, but their data were removed for high error rates (mean accuracy rate >80%). Table 1 shows the participants’ demographic characteristics, visual acuity, and cognitive functioning.

**Table 1:** **Means and standard deviations of participants’ demographic information and results from perceptual and cognitive tests**.

Variable	Younger		Older	
	*M*	SD	*M*	SD
**EDUCATION (%)**				
4-Year college degree or more	15.79		91.30	
Some college	25.00		1.45	
Completed high school only	51.32		7.25	
Some high school or less	7.89		0.00	
**HEALTH**				
Self-rating of health	3.96	0.77	3.72	0.91
**VISION MEASURES**				
Rosenbaum near vision*	23.36	3.86	30.43	5.54
Pelli-Robson contrast*	1.53	0.13	1.40	0.13
**COGNITIVE MEASURES**				
MMSE*	29.68	0.57	28.97	1.01
Digit symbol substitution*	72.11	0.09	53.54	11.16
Shipley vocabulary test*	14.20	9.81	16.56	2.39
**AFFECT MEASURES**				
PANAS positive affect*	29.07	8.78	34.09	5.74
PANAS negative affect*	16.27	5.31	13.83	5.97
**ATTENTION NETWORK TASK**				
ANT alerting effect (RT in ms)*	42.32	26.72	23.64	38.15
ANT orienting effect (RT in ms)	41.88	22.31	46.07	33.06
ANT conflict effect (RT in ms)	134.29	55.14	148.23	93.47
ANT mean accuracy (percentage correct)	98.00	5.81	97.04	4.37

The tests used were as follows: self-reported current health, ranging from 1 (poor) to 5 (excellent); Rosenbaum pocket vision screener for near vision (Rosenbaum, 1984); Pelli-Robson contrast sensitivity chart (Pelli et al., 1988); mini-mental state examination (MMSE; Folstein et al., 1975); digit symbol substitution, a subtest of the Wechsler adult intelligence scale – revised (Wechsler, 1981); Shipley vocabulary test (Zachary, 1986); positive and negative affect schedule (PANAS; Watson et al., 1988); attention network task (ANT; Fan et al., 2002). All participants had a MMSE score of 27 or higher. RT, response time. *Significant age differences at *p* < 0.05.

### Measures

#### Affect

The Positive and Negative Affect Schedule (PANAS; Watson et al., 1988) was used to assess the participant’s current emotional experiences (or states). The PANAS is a 20-item, self-rated measure comprised of two independent dimensions of PA and NA. It consists of 20 adjectives reflecting of positive (10 items, e.g., “interested,” “alert,” “excited,” and “enthusiastic”) and negative (10 items, e.g., “guilty,” “upset,” “distressed,” and “irritable”) affect. Respondents are asked to rate the extent to which they have felt this way in the indicated time frame (*today* for the current study) on five-point Likert-type scales. The scale points are: 1 – “very slightly or not at all,” 2 – “a little,” 3 – “moderately,” 4 – “quite a bit,” and 5 – “extremely.” The PANAS is the most widely used scale to measure current affective states (Crawford and Henry, 2004). Watson et al. (1988) reported alpha coefficients for various time-frames ranging from 0.86 to 0.90 for the PA scale and 0.84 to 0.87 for the NA scale. Test-retest reliability for an 8-week period ranged from 0.47 to 0.68 for PA and from 0.39 to 0.71 for NA. Crawford and Henry (2004), in a study involving a large sample drawn from the general adult population (18–91 years), obtained similar reliability scores of both scales to those found in Watson et al. (1988) and demonstrated the construct validity of the PANAS scales. The reliabilities for the PANAS found in the current study were high in both our younger and older samples (younger adults: α_PA_ = 0.92 and α_NA_ = 0.82; older adults: α_PA_ = 0.83 and α_NA_ = 0.93).

#### Attention

**Figure 1:**
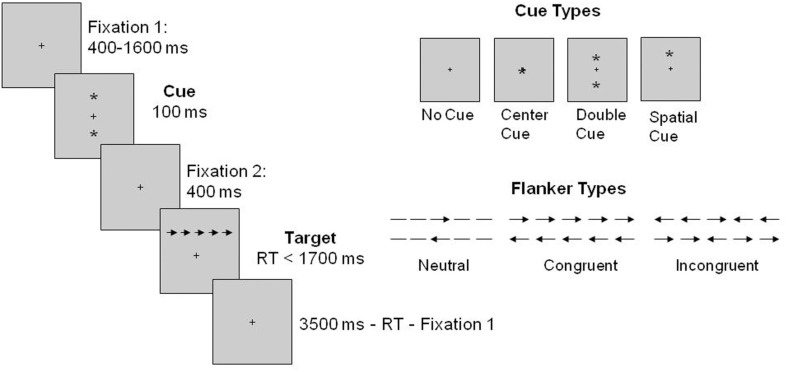
**Sequence of events for each trial of the ANT based on Fan et al. (2002)**.

Figure 1 illustrates the sequence of the ANT. In the ANT, a fixation cross of variable duration (400–1600 ms) was presented at the beginning of each trial. This was followed by one of four cue conditions: no cue, center cue, double cue, and spatial cue. The cue (i.e., an image of an asterisk) was presented for 100 ms. On no cue trials, no cue appeared, but a fixation cross was presented; on center cue trials the cue occurred at the location of the fixation cross; on double cue trials the cues appeared above and below the fixation cross; and on spatial cue trials the cue appeared in the same location as the upcoming target, thus it always predicted the target location. After the cue disappeared, another fixation period of 400 ms was provided and a target-flanker display appeared above or below the fixation cross until the participant gave a response, but for no longer than 1700 ms. The target stimulus (i.e., central arrow pointing left or right) was surrounded by flankers on each side and there were three flanker types: congruent (arrows pointing the same direction as the target, central arrow), incongruent (arrows pointing the opposite direction of the target arrow), and neutral (dashes). After the response was given, the last fixation period was presented (3500 ms minus the first fixation period minus target RT). The total duration for each trial was 4000 ms. The JAVA version of the ANT (downloaded from Fan J’s website) was used in the current study.

### Procedure

Participants were tested individually. After providing informed consent, participants completed a demographic questionnaire and measures of visual acuity, cognition, and affect, followed by the ANT. In the ANT, participants were instructed to press a key indicating the direction of a centrally presented target arrow (pointing either left or right) that appeared above or below a fixation cross, shown in the center of the screen. The ANT began with 24 practice trials (with feedback following errors), followed by three experimental blocks (96 trials/block without feedback) separated by a short break. Participants were instructed to maintain their fixation at the fixation cross all the time, to identify the direction of the target arrow, and that quick and accurate responses would be important.

## Results

### Preliminary analyses

Table 1 depicts mean group differences in the PANAS and the ANT assessed using one-way analysis of variances (ANOVAs) between younger and older groups. For age differences in the PANAS, the older group scored significantly higher on the PA scale compared to the younger group, *F*(1, 143) = 16.25, *p* < 0.001, but scored lower on the NA scale, *F*(1, 143) = 6.81, *p* < 0.05. Thus, consistent with numerous previous findings (e.g., Mroczek and Kolarz, 1998), older adults, on average, reported experiencing more positive states and less negative states than their younger counterparts.

Regarding the ANT performance, the JAVA-ANT provides precalculated scores for each network. The efficiency of each attentional network is calculated using subtractions to determine the influence of alerting cues, orienting cues, and flankers on RTs. The alerting effect is calculated as *RT*_no cue_ − *RT*_double cue_. The orienting effect is calculated as *RT*_center cue_ − *RT*_double cue_. The conflict effect (i.e., executive attention) is defined as *RT*_incongruent_ − *RT*_congruent_. Higher scores on the alerting and orienting effects are indicative of faster cue-related performance [i.e., faster RTs due to warning (alerting) and spatial (orienting) cues]. Higher scores on the conflict effect are indicative of slower RTs due to incongruent flankers (i.e., the costs associated with conflict resolution).

One sample *t*-tests were used to examine whether calculated network scores were different from zero and the results indicated that all the network scores were significantly different from zero (all *p*s < 0.001) for both younger and older adults. Thus, each network score for both age groups provides a usable index of efficiency of each network. Next, age differences in the ANT performance were tested and the results revealed a significantly reduced alerting effect in the older compared to the younger group, *F*(1, 143) = 11.83, *p* < 0.01 (see Table 1), and equivalent orienting and conflict effects in both groups, *F* < 1, and *F*(1, 143) = 1.22, *p* = 0.27, respectively. In order to control for the possibility that age-related declines in speed of processing (Salthouse, 1991) influenced the age difference in the alerting effect, the alerting effect as a function age was re-tested while controlling for age differences in speed. The scores on the Digit Symbol Substitution test (Wechsler, 1981), which indeed revealed age-related slowing in processing speed (see Table 1), were used as a covariate. The age differences in the alerting effect remained significant after adjusting for age differences in speed. As shown in Table 1, there were no age differences in the mean ANT accuracy, indicating accuracy was equally high for both younger and older adults, *F*(1, 143) = 1.23, *p* = 0.27.

Next, Table 2 presents the correlations between the study’s main variables (attention network scores, PA, and NA) in the two age groups. Within the ANT, there were no significant correlations among alerting, orienting, and executive attention for both age groups (e.g., Fan et al., 2002). In the younger group, PA was inversely associated with alerting efficiency, whereas NA was positively associated with orienting efficiency. In the older group, neither PA nor NA was associated with alerting efficiency; however, PA was positively related with orienting efficiency, whereas NA tended to be inversely associated with orienting efficiency. Neither PA nor NA was associated with conflict resolution efficiency (i.e., executive attention) for both age groups.

**Table 2:** **Correlations between the study’s main variables in the two age groups**.

	PA	NA	Alerting	Orienting	Conflict
PA	1	−0.13	−0.13	0.29*	0.10
NA	−0.10	1	−0.02	−0.23†	0.07
Alerting	−0.24*	0.08	1	0.07	−0.17
Orienting	0.02	0.23*	−0.17	1	0.04
Conflict	−0.10	−0.12	0.08	−0.03	1

Correlations below the diagonal are from the younger age group. Correlations above the diagonal are from the older age group. †*p *< 0.07, *p < 0.05.

### The moderating role of age

To test whether the effects of PA and NA on attentional networks were moderated by age, we performed moderated regressions analyses. With each attentional network as a dependent variable, two hierarchical multiple regression analyses were conducted separately for PA and NA. Thus, a total of six regression models were conducted. In the first step of the regression analyses, we included gender (0 = male) and speed of processing as control variables, because age groups had a higher percentage of women, and older adults, on average, were slower in their processing speed than younger adults. In the second step, we tested the main effects of age (0 = younger adults) and PA or NA for significance. Finally, we entered the interaction terms between age group and PA or NA in the last step of the analyses. All continuous predictor variables, except for gender and age, were standardized prior to performance of analyses.

The results of the main effects and interaction effects are reported in Table 3. Across all regression models, gender failed to account for the efficiency of attentional networks, and speed only accounted for alerting efficiency in that slower speed of processing predicted reduced alerting efficiency. For the alerting model with PA, age did not significantly predict alerting efficiency; however, higher levels of PA were associated with lower alerting efficiency. Despite this association, the age by PA interaction was not significant. For the alerting model with NA, the main effect of age was significant as older adults exhibited lower alerting efficiency than younger adults. Neither the main effect of NA nor the age by NA interaction was significant. Thus, the alerting models indicated that age did not moderate the effects of PA and NA on alerting efficiency.

**Table 3:** **Hierarchical regression analyses predicting attentional networks from age group, PA, NA, and the age group by PA/NA interactions**.

	Δ*R*^2^	β		Δ*R*^2^	β
**ALERTING**					
Step 1	0.04				
Gender		−6.54			
Speed		5.74*			
Δ*F*-value		*F*(2, 142) = 2.86†		
Step 2	0.07			0.04	
Age		−13.13	Age		−18.05*
PA		−6.65*	NA		0.99
Δ*F*-value		*F*(2, 140) = 5.73*	Δ*F*-value		*F*(2, 140) = 3.08
Step 3	0.00			0.00	
Age PA		−0.65	Age × NA		−3.13
Δ*F*-value		*F*(1, 139) < 1	Δ*F*-value		*F*(1, 139) < 1
**ORIENTING**					
Step 1	0.01				
Gender		3.01			
Speed		−2.09			
Δ*F*-value		*F*(2, 142) < 1		
Step 2	0.02			0.00	
Age		−2.06	Age		1.02
PA		4.56†	NA		−1.36
Δ*F*-value		*F*(2, 140) = 1.68	Δ*F*-value		*F*(2, 140) < 1
Step 3	0.04		Step 3	0.05	
Age × PA		12.26*	Age × NA		−12.64**
Δ*F*-value		*F*(1, 139) = 5.14*	Δ*F*-value		*F*(1, 139) = 7.15**
**CONFLICT**					
Step 1	0.01				
Gender		2.74			
Speed		−7.96			
Δ*F*-value		*F*(2, 142) < 1		
Step 2	0.00		Step 2	0.01	
Age		4.88	Age		5.52
PA		<1	NA		<1
Δ*F*-value		*F*(2, 140) < 1	Δ*F*-value		*F*(2, 140) < 1
Step 3	0.01		Step 3	0.01	
Age × PA		17.57	Age × NA		14.29
Δ*F*-value		*F*(1, 139) = 1.36	Δ*F*-value		*F*(1, 139) = 1.19

Gender was coded as 0 (male) and 1 (female). Age coded as 0 (younger adults) and 1 (older adults). PA, positive affect; NA, negative affect. †*p *< 0.07, *p < 0.05, and **p < 0.01.

**Figure 2:**
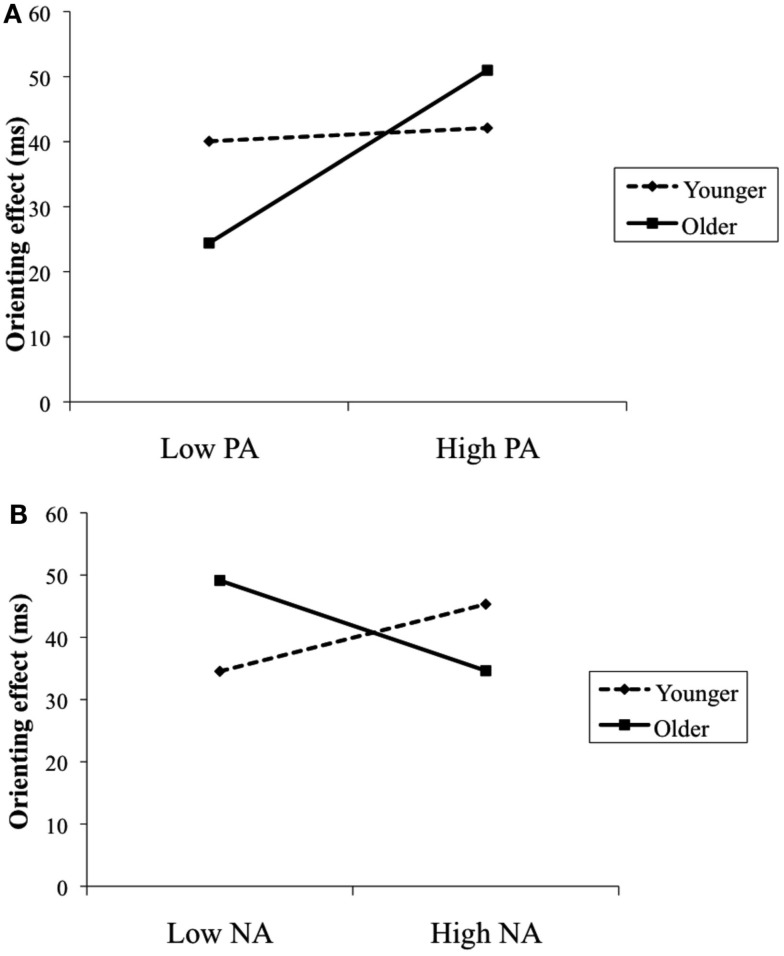
**Age as a moderator of the relationship between PA and orienting effect (A) and the relationship between NA and orienting effect (B)**. Results were controlled for gender and speed.

For the orienting model with PA, neither age nor PA predicted orienting efficiency; however, there was a significant age by PA interaction. In addition, for the orienting model with NA, none of the main effects were significant; though, the age by NA interaction effect was significant. To illustrate the obtained interaction effects, we plotted in Figure 2 the association between PA and NA (1 SD above and below the sample mean) and orienting efficiency separately for younger and older adults employing commonly used regression techniques (Aiken and West, 1991). The obtained pattern of results in Figure 2A indicated that the largest orienting effect was found among older adults with higher levels of PA. A calculation of the simple slopes further supported this interpretation: experiencing higher levels of PA significantly associated with greater orienting efficiency among older adults (β = 13.29, *p* < 0.05), but not among younger adults (β = 1.02, *p* = 0.73). Conversely, the obtained pattern of results in Figure 2B showed that larger orienting effects were found among older adults with lower levels of NA and younger adults with higher levels of NA. A calculation of the simple slopes, however, indicated that the slope for older adults was significant (β = −7.25, *p* < 0.05), but not for younger adults (β = 5.39, *p* = 0.12). Thus, experiencing lower levels of NA was significantly associated with greater orienting efficiency among older adults. Finally, for the conflict models, neither PA nor NA predicted conflict resolution. Moreover, age did not exert a significant interaction with PA or NA on conflict resolution.

### The mediating role of state affect

To determine whether age differences in PA and NA accounted for age differences in alerting, we performed a mediation analysis, using age and alerting as independent and dependent variables and affect variables as mediators. While recent work has suggested important problems with meditational analyses using cross-sectional aging data when the mediator is supposed to represent a developmental process (see Lindenberger et al., 2011), we considered PA and NA as potential *states* that could mediate the relationships of interest, not as developmental outcomes themselves. Thus, meditational analyses may still be appropriate in this context.

The analysis of mediation effects used a multiple mediation model with both PA and NA entered simultaneously using Preacher and Hayes’ (2008) method for testing mediation. By testing for mediation effects through both PA and NA, we were able to compare the size of each effect as well as its significance as a mediation effect. Following Preacher and Hayes’ (2008) method, we used a bootstrapping procedure to compute SEs and 95% confidence intervals around the indirect effect (i.e., the effect of age on alerting through PA and NA). This method uses 5,000 bootstrapped samples to estimate the bias-corrected and accelerated confidence intervals. Indirect effects were considered as significant when the confidence interval did not include zero. The SPSS macros that Preacher and Hayes provided were utilized for this procedure. In this analysis, age (0 = younger adults) was the independent variable, alerting was the dependent variable, and PA and NA were mediators. Preacher and Hayes (2008) recommended use of bootstrap SEs and confidence intervals over the Sobel test because the latter involves the assumption of the normality of the estimates of the indirect effect, which is normal only in large samples. However, for convenience, we also report the traditional mediation significance test by the Sobel test. In addition, unstandardized regression/path coefficients were reported in the current model because standardized coefficients for a dichotomous variable (i.e., *age*) had no meaningful interpretation (Preacher and Hayes, 2008). Significance tests of the mediation effect can be found in Table 4.

**Table 4:** **Magnitude and confidence intervals of the multiple mediation test of the relationship BETWEEN age and alerting through PA and NA**.

	Bootstrap results for mediation effects			
	Mediation effect	SE	95% confidence interval	
			Lower	Upper
**MEDIATORS**				
Total mediated effect	**−5.46**	3.36	−13.89	−0.30
PA	**−5.29**	2.78	−12.85	−1.30
NA	−0.17	1.66	−5.15	2.27
**CONTRAST**				
NA-PA	**−5.11**	3.12	−12.79	−0.15

*Boldface type highlights a significant effect as determined by the 95% bias-corrected and accelerated confidence interval based on 5,000 bootstrap samples*.

**Figure 3:**
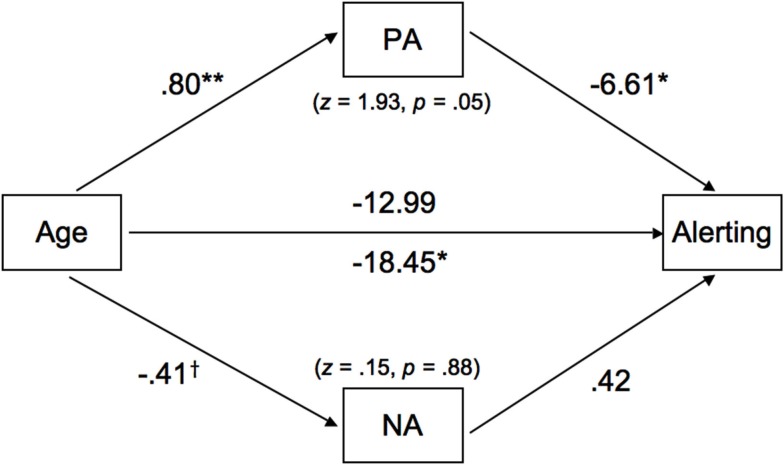
**The mediated roles of positive affect (PA) and negative affect (NA) on the age-alerting association**. Path coefficients are unstandardized beta weights while controlling for gender and speed. The *z*-values (and associated *p*-values) refer to Sobel test results, assessing the significance of the role of each moderator. Two direct effects of age on alerting are included. The coefficient below the path is representative of a model that did not include any mediators; the coefficient above the path is with PA and NA as mediators. Results were controlled for gender and speed of processing. †*p *< 0.07, *p < 0.05, and **p < 0.01.

The total mediation effect of PA and NA was significant; however, this effect was driven by PA, as the mediation effect of NA did not attain significance. Although the result of the Sobel test was only marginally significant (see Figure 3), the fact that zero fell outside the confidence interval (−12.89 to −1.30; see Table 4) indicated a significant mediation effect (Preacher and Hayes, 2008). As can be seen in Figure 3, older adults were more likely to report a higher state of PA (*b* = 0.80, *p* < 0.01), which had a negative prediction effect for differences in alerting efficiency (*b* = −6.61, *p* < 0.05). At the same time, age was a significant predictor of alerting without the mediators in the model (*b* = −18.45, *p* < 0.05), but the direct effect became non-significant in the presence of the mediators, suggesting that PA largely mediated the age differences in alerting.

## Discussion

The current study investigated age differences in the links between affect and attentional functioning. From preliminarily analyses on mean differences, we found that older adults in the current study reported experiencing more PA and less NA than did younger adults. Thus, the finding is consistent with previous studies of age-related decreases in experience of NA (e.g., Mroczek and Kolarz, 1998; Carstensen et al., 2011), which suggest that as people get older, they report more positive and less negative affective states. With regard to age differences in the efficiency of attentional networks, older adults were found to show decreased alerting efficiency compared with younger adults (even when adjusting for speed of processing), but there was no evidence of age differences in the efficiency of orienting and executive attention. These results are consistent with previous findings (Festa-Martino et al., 2004; Fernandez-Duque and Black, 2006; Jennings et al., 2007; Gamboz et al., 2010). Noradrenergic transmission has been linked to alerting (Fernandez-Duque and Posner, 2001; Fan et al., 2009), as well as associated with NA (Sullivan et al., 1999), suggesting that age-related declines in the noradrenergic activation system (Jennings et al., 2007) might explain the main effect of age on both alerting and NA.

Results from the correlation analysis showed no association among the three network scores for both younger and older age groups, indicating the functional independence of alerting, orienting, and executive attention (e.g., Fan et al., 2002; Moriya and Tanno, 2009). Younger and older adults showed somewhat different patterns of correlations between affect and attentional networks, which were further examined by the moderation and mediation analyses.

### Age differences in the association between affect and alerting

Higher PA was associated with diminished alerting, but age group did not moderate the relationship between PA and alerting. This finding is inconsistent with our hypothesis and divergent from previous work linking NA and alerting (e.g., Pacheco-Unguetti et al., 2010; Jiang et al., 2011; Lyche et al., 2011). It is perplexing why PA, not NA, was related with alerting functioning in the current study. If PA broadens attention and thinking (Fredrickson, 1998, 2001), which is hypothesized to induce loosened control of attention (Biss and Hasher, 2011), PA could also modulate the alerting network in similar ways. It has been shown that the processes involved in alerting and executive attention share a common brain region in the frontal-parietal lobe, though the two processes are dissociable at the behavioral level (Fan et al., 2007). The executive attention system was associated with dopaminergic transmission (Fan et al., 2009), which was associated with PA (Ashby et al., 2002). Together, the findings provide indirect evidence that PA may also be related to alerting. However, given no evidence for the link between PA and executive attention in the current study, it seems to be unlikely that high PA is also linked to impaired alerting. The reduced alerting effect can also be interpreted as the facilitated ability to prepare and sustain alertness in the *absence* of a warning cue. Therefore, it may be more plausible, however speculative, that diminished alerting associated with PA indicates higher vigilance and, therefore, an increased ability to maintain alertness in the *absence* of a warning cue.

The results from the mediation analysis further indicate that the majority of the age-related variance in the alerting effect was associated with PA. PA fully mediated age differences in alerting. The present finding may, in part, align with the earlier finding of Phillips et al. (2002b) that older adults in both positive and negative mood states showed worse performance on a task of executive attention than younger adults. It is interesting that we observed this pattern in alerting performance. The present finding, in conjunction with Phillips et al.’s (2002b), may suggest that increased PA is more likely to influence attentional functioning for older adults than for younger adults.

Although we predicted that older adults with higher NA would be more alert that those with lower NA, as they might be more motivated to regulate emotion, we found no supporting evidence for this link. Thus, SST does not seem to be a fitting explanation for this pattern. There may be several tentative explanations for the association between PA and alerting in older adults. One possibility is that, as we speculated above, if the effect of PA on alerting reflects better ability to prepare and sustain alertness in the absence of warning cues, the present result may suggest that high PA could enhance alertness in the absence of warning cues for older adults. Another possibility is that, as we speculated above, if the effect of PA on alerting reflects greater vigilance in the *absence* of warning cues, the present result may suggest that high PA could enhance alertness when warning cues are *not* present. An underlying mechanism for this greater alertness may be related to dopaminergic and norepinephrinergic pathways from the amygdala (LeDoux, 2007 for review of afferent amygdala input) which could be affected by levels of PA. We should caution that this interpretation is speculative and PA actually may be related to worse alerting. In addition, more work is needed in order to determine how the relationship between age and PA influences the neural functioning that is implicated in alerting.

The lack of NA effect on alerting can be explained, in part, by the fact that the NA state measured in the current study may not be strong enough to generate findings similar to those measured in previous studies. For example, previous studies either selected individuals with extreme NA states, such as depression (Lyche et al., 2011), or experimentally manipulated negative mood (Pacheco-Unguetti et al., 2010; Jiang et al., 2011). The current study, on the other hand, assessed naturally occurring individual differences in NA in healthy participants. The arousal levels involved in these varying NA states may differ (Jefferies et al., 2008), though the possibility of this phenomenon does require further investigation. In the current study, the average level of NA reported by the participants was quite low (as indicated in Table 1). This might have resulted in the relatively lower level of arousal of NA found as compared to previous studies. Future studies should address how different ranges of NA states, as well as levels of arousal, may be related to the function of alerting, and how these factors interact with one another to influence the function of alerting, as well as age differences in the NA-alerting link.

### Age differences in the association between affect and orienting

Age group did moderate the relationship between PA and orienting as well as NA and orienting. That is, older adults experiencing higher PA and lower NA exhibited better orienting followed by spatial cues than those experiencing lower PA and higher NA. On the other hand, orienting efficiency among younger adults was not significantly influenced by the levels of PA and NA, although the results of the correlations indicate that NA was positively associated with orienting for younger adults (Pacheco-Unguetti et al., 2010). Thus, supporting our hypothesis, affect states were more likely to influence orienting for older adults. Though our hypothesis was supported, we particularly expected that higher NA would produce more efficient orienting in older adults as previous studies demonstrated pronounced gaze preferences toward positive stimuli in a negative mood state among older adults (e.g., Isaacowitz et al., 2008); however, the pattern in the data showed that older adults experiencing more positive and less negative affective states oriented attention to the cued location much more quickly. This finding conflicts with Compton et al.’s (2004) reports that individuals with low PA showed greater spatial cueing effects than individuals with high PA. As Compton et al. (2004) argued, if greater spatial cueing effects associated with low PA are due to decreased cognitive flexibility associated with low PA, the present finding with older adults may also reflect decreased cognitive flexibility associated with high PA and low NA among older adults. However, as high PA has been closely linked to increased cognitive flexibility (Ashby et al., 1999), this may not be the case. It is noteworthy that there are some methodological differences between Compton et al. (2004) and the current study. Compton et al. (2004) only included younger participants and their orienting task included both valid and invalid cue trials, while the ANT task used in the current study only included valid cue trials. Therefore, the present finding indicates that older adults with high PA and low NA benefited more from valid cues in orienting attention, but cannot address the impact of invalid cues. Future studies should further test whether older adults with high PA or low NA also have difficulty disengaging attention from invalidly cued locations. This may help elucidate whether or not the orienting effect associated with high PA and low NA in older adults reflects decreased cognitive flexibility.

The orienting network is governed by two neural mechanisms: the dorsal frontospatial network (DAN) and ventral frontoparietal network (VAN; Corbetta and Shulman, 2002). DAN is involved in top-down control of directing attention to the location-directing cue and making eye-movements to sensory stimuli, while VAN is involved in disengagement of attention when invalid cue is presented so attention has to be shifted away from its current focus toward the opposite visual field (Corbetta and Shulman, 2002). Because invalid cue trials were not included in the ANT in the current study, the present orienting effect may be related to DAN. However, it is proposed that DAN and VAN interact in a way that DAN provides a signal to VAN in order to maintain a certain attentional balance between the two systems when focusing attention (Corbetta et al., 2008). The present results, therefore, may indicate this orienting system remains balanced in younger adults and is somehow disrupted in older adults by high PA and low NA states. Further, both networks rely on cholinergic transmission (Fan et al., 2009). A deficit in cholinergic transmission in Alzheimer’s disease produces enhanced benefit from valid cues (Parasuraman et al., 1992). Therefore, the present orienting effects could also reflect cholinergic transmission associated with high PA and low NA, which may affect activity in DAN during engagement of attention to valid cues. This possible link needs more investigation.

Alternatively, our finding may indicate that older adults with high levels of PA states may seek out more PA in an effort to perpetuate an “upward spiral” of positive emotion (Fredrickson and Joiner, 2002). These individuals may find more positive information in the world around them, and may maintain greater happiness because of what they find, which could lead them to rely on environmental cues to orient their attention. This possibility might be supported by a recent study by Waldinger et al. (2011). They showed that older adults high in life satisfaction displayed increased connectivity of within an amygdala-mediated network in response to positive information, as compared to negative information. In line with Waldinger et al. (2011), our findings may suggest that older adults reporting high levels of PA states may be more sensitive to environmental cues to orient attention. However, it remains important to test whether individual differences in affective states in older adults are related to orienting attention, and more specifically to emotionally valenced information.

### Age differences in the association between affect and executive attention

There is no evidence from the present results that PA had any effect on executive attention. Thus, the present finding is not in line with the Broaden-and-Build Theory of positive emotions (Fredrickson, 1998). Martin and Kerns (2011) suggest that PA is related to some aspects of executive attention, such as working memory and planning, but it may have little impact on prepotent response inhibition, which is related to flanker interference. However, Rowe et al. (2007) found supporting evidence for the Broaden-and-Build Theory using the flanker task. The discrepancy between the result of this study and that of Rowe et al. (2007) may lie in a difference in the flanker task, as Rowe et al. (2007) manipulated the distance between the flankers and target, while the current study had no such manipulation. Consistent with the present finding, previous studies using the ANT also reported a lack of association between PA and flanker interference (Jiang et al., 2011; Lyche et al., 2011; Martin and Kerns, 2011; cf., Rowe et al., 2007). Thus, future research should, therefore, not only consider different executive functions, but also take into account variations of the flanker task.

Inconsistent with our hypothesis and Phillips et al.’s (2002b) results, we found no evidence for diminished executive attention among older adults in either PA or NA states. The discrepancy between our finding and those of Phillips et al. (2002b) may lie in the task used to assess executive attention and the way affect was measured. Such methodological differences may cause different results. Therefore, future research should be conducted to examine whether the influence of affect on executive attention is dependent upon how executive attention and affect (or mood) are assessed.

### Limitations and conclusion

The current study has several important limitations that are important to note. Most importantly, the data in the current study come from self-reported measures of affect and from non-clinical participants. Thus, the data may reflect restricted ranges of affect that may not be sensitive to detect possible affect-attention relationships. In addition, because the context of emotion regulation was not directly manipulated in the current study, how age-related changes in affective experiences and shifts in emotional goals interact to influence attentional functioning remains in question. This limitation could be attributed to the fact that we found no evidence for our hypotheses regarding the attentional performance of older adults with high NA. Future research will need to make explicit the connections between age differences in affect-attention links as a function of emotional goals. Further, this study is cross-sectional and does not include middle-aged participants, which precludes a direct examination of age-related changes in affect and attentional networks and how these changes influence one another. Finally, although the current study provides initial behavioral evidence regarding age differences in the effects of PA and NA on attentional networks, future brain-imaging studies will shed further light on the interactive processing of affect and attentional networks at a neural level.

These limitations notwithstanding, the results presented here show that attention is indeed a multifaceted construct and each facet can be affected differently by PA and NA. Additionally, individual difference factors such as age should continue to be taken into account when researchers study the affect-attention relationship. Extending the previous studies showing age differences in attentional orienting to emotional information, the present results indicate that age differences in alerting and orienting can be modulated by state PA or/and NA when the task is non-emotional. Experiencing high positive and low negative affective states may be adaptive in later years of life. The present results that such positive affective profile is more likely to influence attention for older adults than younger adults suggest that this relationship may occur in order to compensate for age-related changes in internal resources. This relationship needs further investigation.

## Conflict of Interest Statement

The authors declare that the research was conducted in the absence of any commercial or financial relationships that could be construed as a potential conflict of interest.
